# An Account of a Species of Cantharis, Found in Buck's County, Pennsylvania; Including Observations on Its Medical Properties

**Published:** 1799-04

**Authors:** Isaac Chapman


### *An Account of a Species of Cantharis, found in Buck's County, Pennsylvania; including Observations on its Medical Properties: By Dr.* Isaac Chapman.

THIS insect has a very near resemblance, in its external form, to the *meloe vesicatorius, alatus viridissimus, nitens, antennis nigris. Linn*—or Spanish flies, as they are commonly called, but is rather smaller than those brought from Spain, and of a different colour; the head is of a very light red, with black antennæ, the elytra, or wing-cases, are black, margined with pale yellow, and a stripe of the same colour extends along the middle; the the tarsi have five articulations; the mouth is armed with jaws, and furnished with palpi.

Observing them plentiful in my garden, I caught as many as weighed about an ounce when dried. Isaac Praul, one of the young gentlemen studying medicine with me, powdered five or six of them, and laid the powder on a plaster, which he applied to his ancle, and in eight or nine hours, they produced a very good blister.

*(Dr. Chapman then enumerates three cases where he applied these flies, in all of which, they drew good blisters:—he thus proceeds)*—

I have since used these cantharides in near one hundred cases, as vesicatories, and, in every trial, I found their qualities equal, and rather superior to those of European cantharides; they appeared to have fully as much effect in relieving the symptoms, and removing the diseases for which they were applied; and their effect on the system was the same, having, in several cases, produced a slight strangury; the diseases in which I used them were various, as fevers, pleurisy, nervous disorders, &c.

From several experiments *(which Dr. Chapman recites)* it appears that every part of the insect is endowed with an equal, or nearly an equal degree of their quality; they likewise shew their great power, as about one quarter of a grain was sufficient to produce a good blister as large as a shilling.

*(Then follows the manner of gathering and drying the insects, which, as it is chiefly interesting to the inhabitants of America, we here omit.*

